# Thymic Hyperplasia after Lung Transplantation Imitating Posttransplant Lymphoproliferative Disorder

**DOI:** 10.1155/2011/859405

**Published:** 2011-09-06

**Authors:** Christina Maria Steger, Severin Semsroth, Thomas Hager, Ralf Rieker, Ludwig Müller

**Affiliations:** ^1^Department of Pathology, Innsbruck Medical University, 6020 Innsbruck, Austria; ^2^Department of Cardiac Surgery, Innsbruck Medical University, 6020 Innsbruck, Austria; ^3^Department of Pathology, University of Erlangen, 91054 Erlangen, Germany

## Abstract

Thymic hyperplasia is usually associated with the treatment of malignant tumours and is sometimes linked with endocrine diseases. For the first time, we report a case of thymic hyperplasia in a patient 2 years after bilateral lung transplantation. Contrast-enhanced chest CT scan was highly suspicious for a posttransplant lymphoma or thymoma. Therefore, the patient received total thymectomy. Excised specimens were sent to the Department of Pathology. Unexpectedly, the histological examination revealed hyperplastic thymic tissue without evidence for a posttransplant lymphoproliferative disorder or malignancy.

## 1. Introduction

Thymic hyperplasia is an increase in the volume of the thymus gland by formation of new cellular elements in a normal microscopic arrangement. Histologically, the thymus gland contains 3 major cell populations: epithelial, hemopoietic cells (with three main morphology distinct types of lymphoid cells) and accessory cells. Epithelial cells promote different steps of intrathymic T-cell differentiation and maturation. Macrophages secrete a thymocyte-differentiating factor that is mitogenic and induces functional maturation of the thymocyte. Interdigitating cells have a role in determining which T-cell precursors are activated (helper or killer) during an immunological challenge. Myoid cells demonstrate acetylcholine receptors and have a possible role in myasthenia gravis, an autoimmune disorder of neuromuscular transmission first recognized by Wilks in 1877 [1].

In general, two morphological types of thymic hyperplasia exist: true hyperplasia and lymphofollicular hyperplasia.

*True thymic hyperplasia* [2] generally occurs in three different clinicopathologic forms.

1. True massive thymic hyperplasia *without association with any other disease* is an extremely rare occasion. The cause is unknown; it may be due to thymic hyperfunction or dysfunction related to the endocrine activity of the gland. Patients usually present with symptoms of irritation of the mediastinal structures and symptoms may range from none to respiratory distress.
2. It appears as a *rebound phenomenon* in a number of conditions like recovery from severe stress situations, after administration of steroids, after remission of Cushing's syndrome, and after treatment of malignant tumors.
3. It appears* in association with endocrine abnormalities* (Graves' disease, acromegaly, thyrotoxicosis, hypothyroidism, and Addison disease), sarcoidosis, and Beckwith-Wiedeman syndrome.

*Lymphofollicular thymic hyperplasia* [3] is characterized by its histological appearance, which is composed of lymph follicles with germinal centers similar to those in medullar lymph nodes of a normal-sized thymus. 

Lymphofollicular thymic hyperplasia has been described in: (1) chronic disseminated infections, (2) endocrinopathies, and (3) autoimmune diseases (myasthenia gravis, systemic lupus erythematosus, scleroderma, rheumatoid arthritis, periarteritis nodosa, Hashimoto's thyroiditis, autoimmune anemia, Behçet disease, ulcerative colitis, and multiple sclerosis).

## 2. Case Presentation

A 28-year-old female patient suffered from mucoviscidosis since childhood. 

Besides, she developed mucoviscidosis-related insulin-dependent diabetes mellitus and recurrent hemoptysis caused by bronchiectasis. Esophageal varices due to a Child class A hepatic cirrhosis were treated by variceal banding.

Due to the severe progression of the disease, the patient was listed for lung transplantation since January 2007 and received bilateral lung transplantation in December 2007. Initial immunosuppressive therapy consisted of daclizumab (an interleukin-2 receptor antagonist), cyclosporine A, mycophenolate mofetil, and prednisolone. In March 2008, the treatment was switched to Prograf (tacrolimus) and prednisolone till July 2008 afterwards to everolimus, cyclosporine and prednisolone. Due to everolimus-induced edemas of the lower extremities, the therapy was changed once again from everolimus to mycophenolate mofetil and prednisolone in January 2009. Since August 2009, the immunosuppressive regime was complemented by cyclosporine A. The immunosuppressive therapy was not interrupted at any point of time.

Three episodes of acute cellular rejection were recognized after transplantation. The first episode occurred 10 days after transplant (A2, B1-2). A further steroid refractory rejection episode (A2) was diagnosed four weeks after transplantation, and treated with alemtuzumab (Campath-1H). The third rejection episode occurred in May 2008. 

**Figure 1:**
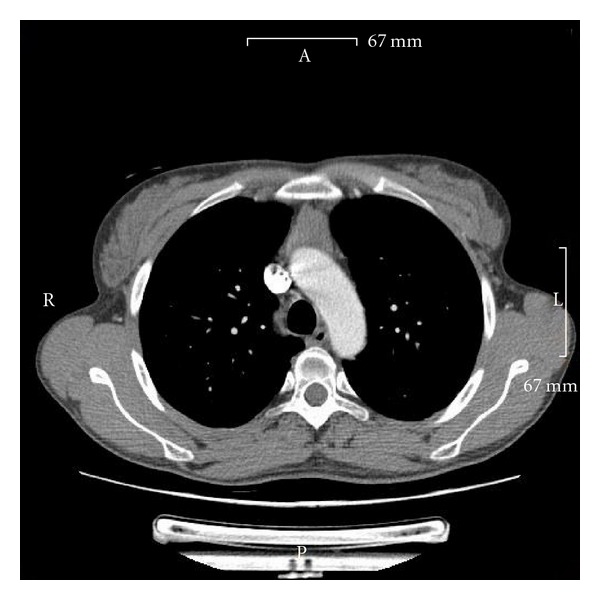
CT scan of the thorax showing the enlarged mediastinal mass in front of the aortic arch.

Contrast-enhanced chest CT scan due to worsening dyspnoea in October 2009 revealed an enlargement of the mediastinum (Figure 1). Radiologically, the enlarged mediastinal mass was highly suspicious of a thymoma or mediastinal posttransplant lymphoma. Clinical laboratory parameters (blood count, LDH, immunoglobulins, and virus titres) were all within the normal range.

Due to the large size of the mediastinal mass, a total thymectomy via hemisternotomy was performed in November 2009. 

Postoperatively, the patient suffered from a supposedly cytotoxic cerebral edema and oculomotorius nerve damage. Currently, the patient is in good general condition.

The resected specimens were sent to the Department of Pathology. Gross examination revealed a 11 : 9 : 1 cm-sized grey-coloured, firm, knotty mass weighing 53 g. 

After fixation, dehydration, and wax embedding, slides of 3 *μ*m thickness were routinely stained with hematoxylin and eosin. 

The mediastinal mass was essentially composed of histologically normal but increased thymic lobules with preserved corticomedullary differentiation, surrounded by mature fat with hyperaemic vessels enclosed. In the middle of the lobules, several enlarged Hassall corpuscles were observed. Adjacent to the thymic tissue three reactive lymph nodes were found. 

**Figure 2:**
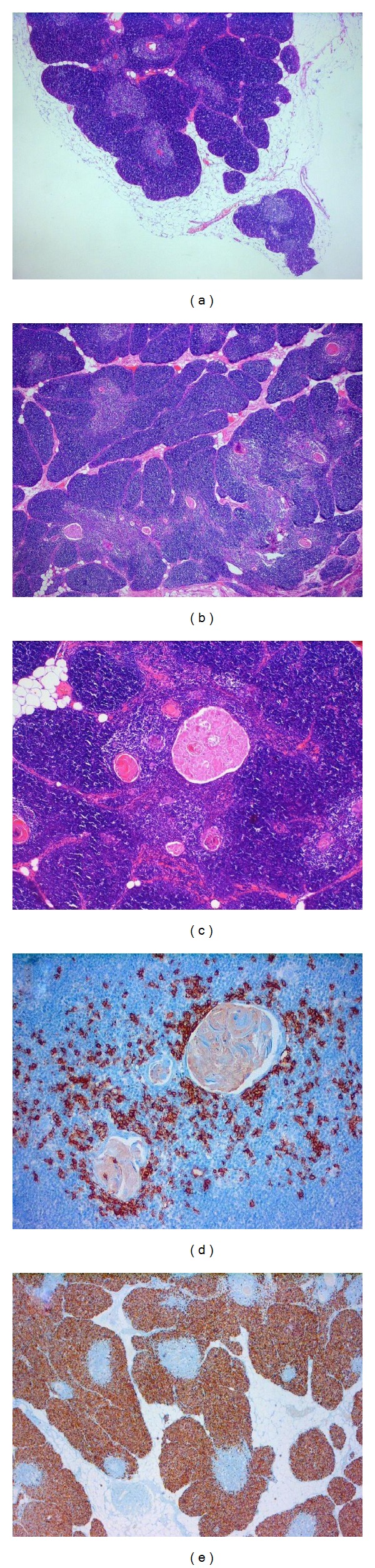
(a) Thymic tissue with adjacent mature fat (hematoxylin and eosin, original magnification ×4). (b) Hyperplastic thymus with lobular structure, divorcing fibrous septa, sometimes interrupted by mature fat and enclosed hyperemic vessels (hematoxylin and eosin, original magnification ×4). (c) Thymic tissue with prominent enlarged Hassall's corpuscles (hematoxylin and eosin, original magnification ×20). (d) Immunohistochemistry shows CD20 positivity for B lymphocytes surrounding Hassall's corpuscles (original magnification ×20, methylen blue counterstain). (e) Strong Ki-67 positivity, which demonstrates the high proliferation rate of the cellular elements of thymic tissue. The staining relieves the areas surrounding the Hassall's corpuscles (original magnification ×4, methylen blue counterstain).

Neither histologically nor immunohistochemically (staining with CD3, CD5, CD20, CD79a, p27, cyclin D1, and Ki-67) the suspicion for a posttransplant lymphoproliferative disease or malignancy could be affirmed (Figure 2).

## 3. Discussion

Thymic hyperplasia is usually described in the stem-cell transplant setting, but it is only reported once before as rebound phenomenon after solid organ transplantation. 

Gerhardt et al. [4] observed a case of thymic hyperplasia occurring 12 years after renal transplantation of a 41-year-old male patient admitted to hospital suffering from acute left chest pain and dyspnoea. At the age of 29 years, he received a renal transplantation due to congenital kidney aplasia which had to be removed a year before admission because of transplant glomerulopathy. Following computed tomography demonstrated a blood-containing mediastinal tumour and a haemothorax. The tumour was resected, and histological examination revealed thymic hyperplasia with extensive bleeding from the thymus. In contrast to our case, this case shows that the termination of immunosuppression can lead to thymic rebound.

A possible explanation for the occurrence of thymic hyperplasia in this case is a noncompliance of the patient concerning the immunosuppression with prednisolone although the patient denied a termination of the corticosteroid therapy. Another possible explanation is the treatment with alemtuzumab although thymic hyperplasia as adverse effect after alemtuzumab treatment was never described before. 

In general, rebound thymic hyperplasia is described in numerous conditions, such as after recovery from severe stress (e.g., burns [5], cardiac surgery [6], after tuberculosis [7], and chemotherapy [8–12]) and has been reported most often in children and adolescents. The mechanism is thymic depletion resulting from high plasma glucocorticoid concentrations [13] followed by rebound thymic hyperplasia when cortisol levels drop [14].

Thymic hyperplasia after cytotoxic chemotherapy may be due to rebound enlargement after initial atrophy caused by chemotherapy. It has been reported mostly in younger age groups and is described in the literature to be associated with various types of cancers, including lymphomas (Hodgkin and non-Hodgkin lymphomas [9, 10]), leukemias, testicular cancer, sarcomas (osteosarcoma [8] and embryonal rhabdomyosarcoma), Wilms tumor [8, 15], and germ cell tumours.

Most cases of rebound thymic hyperplasia secondary to successful chemotherapy (especially in lymphoma or testicular carcinoma) occur within a year, and the gland typically returns to normal size [16]. Pathologically, the rebound thymus hyperplasia is composed of the normal proportions of glandular/lymphoid elements in an enlarged gland. The thymus can grow more than 50% after chemotherapy or illness. All reported cases of thymic rebound were detected on routine chest radiograph with no other clinical or laboratory positive findings.

For successful therapy, it is important to differentiate thymic hyperplasia from residual lymphoma or other tumours of the thymus. But anterior mediastinal masses following chemotherapy for malignant disease often cause diagnostic problems, and differential diagnosis of thymic hyperplasia from recurrence frequently poses a challenge both for the radiologist and the physician. Typically, chest radiograph and CT scan show a widening of the mediastinum. However, this radiological feature allows only a tentative diagnosis which must be confirmed by histological investigation of a biopsy or resected tissue.

Compared with thymic rebound, true massive thymic hyperplasia without any discernable cause is also a rare disease with fewer than 50 recorded cases. Most cases occur between the ages of 1 and 15 years. The next common age group is less than 1 year at age or younger. True massive thymic hyperplasia occurs twice as often in boys than in girls. About 85% of patients are symptomatic with cough, shortness of breath, respiratory distress, and pulmonary infection as most common clinical symptoms. Chest pain has been observed but is unusual. Lymphocytosis in the peripheral blood is seen in about one-fourth of patients. 

Surgical excision via median sternotomy, clamshell incision, or even a unilateral posterolateral thoracotomy is required in all cases in which the organ causes airway obstruction or upper venous obstruction.

In conclusion, in patients with a newly recognized anterior mediastinal mass, most of the alternative diagnosis possibilities should have been ruled out by appropriate clinical and laboratory examinations. A brief course of steroids could be attempted if a corticosteroid-sensitive tumour could be excluded. Steroids often shrink a hyperplastic thymus. Lymphomas and leukemias, however, may be equally responsive to steroids. If the steroid test is inconclusive, a diagnostic mediastinoscopy is necessary. In case of symptomatic or calcified mass, complete resection is needed to make the diagnosis and to obviate malignancy.
